# Hearing Loss among Children Visiting Department of Otolaryngology and HNS of a Tertiary Care Centre

**DOI:** 10.31729/jnma.8326

**Published:** 2023-11-30

**Authors:** Preeti Chaudhary, Ganesh Bahadur Chalise, Arun Adhikari, Luna Mathema, Prasanta Poudyal, Bijay Khatri

**Affiliations:** 1 Department of Otolaryngology & HNS, B.P. Eye Foundation, Hospital for Children, Eye, ENT, and Rehabilitation Services, Madhyapur Thimi, Bhaktapur, Nepal; 2 Academic and Research Department, B.P. Eye Foundation, Hospital for Children, Eye, ENT, and Rehabilitation Services, Madhyapur Thimi, Bhaktapur, Nepal

**Keywords:** *audiology*, *audiometry*, *hearing loss*, *outpatients*, *prevalence*

## Abstract

**Introduction:**

Hearing loss is defined as the partial or total reduction in auditory acuity. Hearing loss can cause detrimental effects on speech, language, developmental, educational, and cognitive outcomes in children. This study aimed to find out the prevalence of hearing loss among children visiting the Department of Otolaryngology and HNS of a tertiary care centre.

**Methods:**

A descriptive cross-sectional study was conducted among children aged 5-19 years visiting the outpatient Department of Otolaryngology and HNS between 1 January 2022 and 31 December 2022 after obtaining ethical approval. All the patients who underwent pure tone evaluation were included in the study. A convenience sampling technique was used. The point estimate was calculated at a 95% Confidence Interval.

**Results:**

Among 3051 children, 328 (10.75%) (9.65-11.85, 95% Confidence Interval) had hearing loss. Among children with hearing loss, 170 (51.83%) of children were female. The mean age of children with hearing loss was 13.31±3.39 years. The mean pure tone average among 452 ears with hearing loss was 44.60±17.71 dB. The commonest degree of hearing loss was mild hearing loss 266 (58.85%), and the commonest type was conductive hearing loss 310 (68.58%). Among children with hearing loss, 124 (37.80%) had bilateral hearing loss.

**Conclusions:**

The prevalence of hearing loss among children visiting the Department of Otolaryngology and HNS was found to be lower than similar studies done in similar settings.

## INTRODUCTION

Globally, hearing loss is common in children, with an estimated 34 million children having deafness or hearing loss, of which 60% of cases are due to preventable causes.^1^ Hearing impairment affects physical health, mental health, and overall quality of life.^2^ Besides, hearing loss can have pernicious effects on speech, language, developmental, educational, social functioning, and cognitive outcomes in children.^3^

Ear and hearing care is the least prioritized area of health care in Nepal, though hearing and speech are the leading causes of disability in Nepal. The estimated prevalence of hearing impairment in the 1990s in children aged 5-15 years was 8.32%, while a study from 2015 to 2019 among school-aged children in Nepal showed 5.73% had hearing impairment.^4,5^

This study aimed to find out the prevalence of hearing loss among children visiting Department of Otolaryngology and HNS of a tertiary care centre.

## METHODS

A descriptive cross-sectional study was conducted among children aged 5 to 19 years visiting the Outpatient Department of Otolaryngology and HNS at the Audiology Unit of the Hospital for Children, Eye, ENT, and Rehabilitation Services (CHEERS), Lokanthali, Bhaktapur, Nepal. The ethical approval was taken from the Ethical Review Board of Nepal Health Research Council (Reference number: 3792). The records of children patients visiting the audiology unit for hearing evaluation following referral from the otolaryngology outpatient clinic between 1 January 2022 to 31 December 2022 were collected from 1 August 2023 to 31 August 2023. All the patients who underwent pure tone evaluation were included in the study. The record review permission was taken from CHEERS. The records available at the Audiology Unit were selected for the study. A convenience sampling method was used. The sample size was estimated using the following formula:


$$\begin{array}{ll} \text{n} & ={\text{Z}}^{2}\times \frac{\text{p}\times \text{q}}{{\text{e}}^{\text{2}}}\\ & =1.96^{2}\times \frac{0.50\times 0.50}{0.02^{2}}\\ & =2401 \end{array}$$

Where,

n = minimum required sample size
Z = 1.96 at a 95% Confidence Interval (CI)
p = prevalence taken as 50% for maximum sample size calculation
q = 1-p
e = margin of error, 2%

The calculated sample size was 2401. However, 3,051 children were included in the study.

All children with complaints or suspicion of hearing loss are sent for hearing tests using pure tone audiometry (PTA). PTA procedure was accomplished in a double-walled, sound-treated audiometric booth, using a two-channel calibrated clinical audiometer (Maico-42) and a supra-aural headphone (Telephonics TDH-49P). Pure tone thresholds were obtained at six-octave band frequencies from 250-8000 Hz, using a 10 decibel (dB) up and 5 dB down regimen according to the modified Hughson-Westlake method.^6^

The degree of hearing loss in this study refers to the severity of hearing loss. It has been categorized into different levels based on the dB range of hearing loss in relation to normal hearing. Mild, moderate, moderately severe, severe, and profound hearing loss was defined as hearing loss between 26 to 40 dB, 41 to 55 dB, 56 to 70 dB, 71 to 90 dB, and more than 90 dB, respectively.^6^ The three types of hearing loss were categorized according to the auditory system affected. The conductive type of hearing loss has damage in the external ear and middle ear, the sensorineural type has damage in the inner ear, and the mixed type of hearing loss has damage in the external or middle ear and the inner ear.^6^

The record review proforma was developed from the literature review and the advice of fellow otolaryngology-HNS Surgeons, Consultant Audiologists, and Public Health experts.

The data collected were entered in Microsoft Excel 2019 and analyzed using IBM SPSS Statistics version 26.0. The point estimate was calculated at 95% CI.

## RESULTS

Among 3051 children, 328 (10.75%) (9.65-11.85, 95% Cl) had hearing loss. Among 328 patients, 124 (37.80%) had bilateral hearing loss. The mean pure tone average among 452 ears with hearing loss was 44.60±17.71 dB. Similarly, the pure tone average in 267 right ears with hearing loss was 44.27±17.14 dB, and in 185 left ears with hearing loss was 45.08±18.55 dB. Among the children with hearing loss, the commonest degree of hearing loss was mild hearing loss (58.85%) (Table 1).

**Table 1:** Distribution of degree of hearing loss among Pediatric patients with hearing loss (n= 452).

Degree of hearing loss	Right ear n (%)	Left ear n (%)	Total n (%)
Mild	153 (33.85)	113 (25)	266 (58.85)
Moderate	66 (14.60)	41 (9.07)	107 (23.67)
Moderately severe	23 (5.09)	13 (2.88)	36 (7.96)
Severe	16 (3.54)	7 (1.55)	23 (5.10)
Profound	9 (1.99)	11 (2.43)	20 (4.42)

Conductive hearing loss was the most common 310 (68.58%) type of hearing loss among children with hearing loss (Table 2).

**Table 2:** Distribution of type of hearing loss among children with hearing loss (n= 452).

Type of hearing loss	Right ear n (%)	Left ear n (%)	Total n (%)
Conductive	188 (41.59)	122 (26.99)	310 (68.58)
Sensorineural	61 (13.50)	46 (10.18)	107 (23.78)
Mixed	18 (3.98)	17 (3.76)	35 (7.74)

Among children with hearing loss, 170 (51.83%) of children were females. The mean age of children with hearing loss was 13.31±3.39 years (Table 3).

**Table 3:** Baseline characteristics of children with hearing loss (n= 328)

Characteristics	n (%)
**Sex**	
Male	158 (48.17)
Female	170 (51.83)
**Age group (years)**	
5-9	52 (15.85)
10-14	141 (42.99)
15-19	135 (41.16)

## DISCUSSION

The prevalence of hearing loss among children in our study was 10.75%. The previous study showed the prevalence to be 12.7%.^7^ An earlier study in the 1990s reported the prevalence of hearing impairment to be 4.52% among children aged 5-15 years in Nepal.^4^ The prevalence of hearing loss in school-aged children conducted at 509 government schools between 2015 to 2019 in Nepal covering all three regions (plains, hilly, and mountainous) was 5.73%.^5^ The study from the early 1990s was a community-based study; the other was a school-based one from the past decade. The study settings of these two studies and our study are entirely different. The higher prevalence in our study can be related to the fact that our study was conducted in a tertiary-level referral otolaryngology hospital with dedicated audiology services, where suspected cases of children with hearing loss or at risk of hearing loss often visit. Nevertheless, the burden indicates that the prevalence of hearing loss among children is a public health problem that needs urgent attention in Nepal.

In our study, more than half (51.83%) of children with hearing loss were females. In a study among school-aged Nepalese Children, the prevalence of hearing loss was slightly higher in males (51.70%).^5^ The slight difference in sex in hearing loss implies that both sexes are equally prone to hearing loss. A study in Karnataka among school-going children showed that among 126 students with hearing loss, 50% were males and 50% were females.^8^ However, in a study among younger children aged 4-7 years with hearing loss in an underserved community in South Africa, 54.2% were females.^9^

In our study, among children with hearing loss, 62.20% had unilateral hearing loss. A similar finding was also observed in a school-based study in Nepal and Kyrgyzstan, where 68.18% and 65%, respectively had unilateral hearing loss among children with hearing loss.^5,10^ However, the study in Karnataka showed that 78.45% of students with hearing loss had bilateral hearing loss.^8^ Studies from South Africa among younger children (4-7 years preschool children and children aged one months to six years old) with hearing loss also showed that bilateral hearing loss was more common.^9,11^ Among children with hearing loss, hearing loss was more common in children aged 10 years or older in our study. The study among school students in Nepal also showed hearing loss increased with age, and similar age groups had a higher prevalence of hearing loss.^5^

In our study, the commonest degree of hearing loss among children with hearing loss was mild hearing loss (58.85%), followed by moderate, moderately severe, severe, and profound hearing loss. A similar pattern was also reported in the study from Nepal.^5^ Mild hearing loss was also the most prevalent among preschool children (4-7 years old) with hearing loss in low-income South African communities.^9^ Among children with hearing loss, 68.58% had conductive hearing loss in our study. This was followed by sensorineural hearing loss (23.78%) and mixed hearing loss (7.74%). This finding is in agreement with another study in Nepal where conductive hearing loss was the most common type of hearing loss observed in a similar proportion (70.47%) of children with hearing loss as in our study, followed by sensorineural hearing loss in 25.68%, and mixed hearing loss in 3.84%.^5^ However, in a study in urban South Africa among 6-12-year-old school-going students with hearing loss, 57.1% had conductive hearing loss, 22.9% had mixed hearing loss, and 20.0% had sensorineural hearing loss.^12^ A study among primary school children aged 6-10 years with hearing loss in the North Arcot District of Tamil Nadu, India, also showed that conductive hearing loss was most common.^13^

Conductive hearing loss among these children can usually be managed by correcting the underlying aetiology, such as treating otitis media with antibiotics or surgically in cases of external ear atresia or ossicular malformations. Mild to moderate sensorineural hearing loss can be managed with hearing aids. Severe to profound bilateral SNHL can be managed by cochlear implantation (unilateral or bilateral) if picked up early, while mild to moderate bilateral SNHL is easier to manage with hearing aids.^14^ However, hearing aids and cochlear implants can be expensive and may not be affordable to every parent in Nepal. Hearing loss among children is a significant health concern, and early detection and intervention can significantly improve outcomes and enhance overall well-being in the immediate and future.

The major limitation of our study is that the study was conducted in a tertiary-level otolaryngology centre with regular audiology services, and the findings may not be generalizable to other community settings or institutional settings.

## CONCLUSIONS

The prevalence of hearing loss in our study was found to be lower than in other studies conducted in similar settings.
